# The HRA2pl fusion peptide exerts *in vitro* antiviral activity against human respiratory paramyxoviruses and pneumoviruses

**DOI:** 10.3389/fcimb.2023.1125135

**Published:** 2023-04-20

**Authors:** Uriel Cruz Meza, Norvell Perezbusta Lara, Laura Chávez Gómez, Marcela Solís Rodríguez, Javier R. Ambrosio Hernández, Rocio Tirado Mendoza

**Affiliations:** ^1^ Department of Microbiology and Parasitology, Faculty of Medicine, Universidad Nacional Autónoma de México (UNAM), Mexico City, Mexico; ^2^ Pharmaceutical Chemistry Department, University of Kansas, Douglas, KS, United States

**Keywords:** acute respiratory infections, human respiratory virus, paramyxovirus, pneumovirus, fusion peptide, syncytium size

## Abstract

Acute respiratory infections are a group of diseases caused by viruses, bacteria, and parasites that mainly affect children until the age of 5 and immunocompromised senior adults. In Mexico, these infections are the main cause of morbidity in children, with more than 26 million cases of respiratory infections reported by the Secretariat of Health, in 2019. The human respiratory syncytial virus (hRSV), the human metapneumovirus (hMPV), and the human parainfluenza-2 (hPIV-2) are responsible for many respiratory infections. Currently, palivizumab, a monoclonal antibody against the fusion protein F, is the treatment of choice against hRSV infections. This protein is being studied for the design of antiviral peptides that act by inhibiting the fusion of the virus and the host cell. Therefore, we examined the antiviral activity of the HRA2pl peptide, which competes the heptad repeat A domain of the F protein of hMPV. The recombinant peptide was obtained using a viral transient expression system. The effect of the fusion peptide was evaluated with an *in vitro* entry assay. Moreover, the effectiveness of HRA2pl was examined in viral isolates from clinical samples obtained from patients with infections caused by hRSV, hMPV, or hPIV-2, by evaluating the viral titer and the syncytium size. The HRA2pl peptide affected the viruses’ capacity of entry, resulting in a 4-log decrease in the viral titer compared to the untreated viral strains. Additionally, a 50% reduction in the size of the syncytium was found. These results demonstrate the antiviral potential of HRA2pl in clinical samples, paving the way toward clinical trials.

## 1 Introduction

Acute respiratory tract infections (ARTIs) represent a persistent public health problem (Lu et al., 2013b) and one of the main causes of morbidity and mortality in both neonates and infants (Yang et al., 2017). These infections range from asymptomatic to moderate and, in some cases, produce a severe infection. ARTIs affect the upper respiratory tract by causing colds, rhinosinusitis, pharyngitis, laryngitis, tracheitis, and otitis media (Schwarze, 2010), and the lower respiratory tract by causing tracheitis, wheezing, bronchitis, bronchiolitis, and pneumonia (Zappa et al., 2008). The latter three are considered the most frequent complications and death causes of ARTIs (Bicer et al., 2013; Perezbusta-Lara et al., 2020b). The etiological agents of ARTIs are primarily bacteria and viruses. Among the most common respiratory viruses are the human respiratory syncytial virus (hRSV), the human parainfluenza type 2 (hPIV-2), and emerging viruses, such as the human metapneumovirus (hMPV) (García-García et al., 2017). Recently, other respiratory viruses have been identified as etiological agents of ARTIs, such as the human bocavirus (Allander et al., 2005), the human coronaviruses NL63 and HKU1, the new human enterovirus, parechovirus, and rhinovirus strains (Berry et al., 2015).

The World Health Organization estimates that approximately 25% of hospitalizations of children are due to ARTIs (Perezbusta-Lara et al., 2020b). hRSV (subgroup A and subgroup B) is responsible for 80% of ARTIs, followed by hMPV with a share of 5–15% (Price et al., 2019; Rodríguez et al., 2020). These agents affect the pediatric population throughout the year (Perezbusta-Lara et al., 2020b). However, from December to February, an increased incidence of respiratory pathologies caused by infections occurs in children younger than 5 years old (Özgür and Demet, 2021). Premature newborns and infants with asthma, congenital heart disease, or bronchopulmonary dysplasia are more susceptible to these infections, usually presenting more severe clinical symptoms compared to children without comorbidities (Shachor-Meyouhas et al., 2011; Manzoni et al., 2017). Moreover, these respiratory infections are relevant in the context of high-risk populations such as the elderly and immunosuppressed patients (Takeuchi and Akira, 2009; Pierangeli et al., 2018).

Unfortunately, effective therapy is not yet available for these viral infections. To date, ribavirin and hRSV immunoglobulin are the only approved drugs for the treatment and prevention of hRSV in high-risk patients (Perezbusta-Lara et al., 2020b). In addition, various monoclonal antibodies are in use for the inhibition of viral entry to cells. Palivizumab is a humanized monoclonal antibody against the hRSV fusion protein F. It is approved as a preventive treatment against serious hRSV disease in infants with specific risk factors (Madhi et al., 2020). Nevertheless, its use has some limitations, including the administration of multiple doses, an efficacy of approximately 45% to 55% among high-risk infants (Madhi et al., 2020), and high costs (Morris et al., 2009). Other monoclonal antibodies against hRSV’s F protein exist, like motavizumab (O’Brien et al., 2015) and nirsevimab (Muňoz et al., 2019). Both underwent clinical trials and demonstrated protective properties against hRSV infection (Madhi et al., 2020). The partial efficacy of the F protein monoclonal antibodies highlights the need to develop new, effective, and safe alternative drugs for the treatment of these infections (Shang et al., 2021).

Antiviral peptides are an alternative to expensive treatments such as monoclonal antibodies (Madhi et al., 2020). In this vein, Guy Boivin’s research group in Canada tested several recombinant fragments of the heptad repeats (HR) A and B (conserved domains of the F protein) of hMPV expressed in *Escherichia coli*. Among them, HRA2 showed the best rates of inhibition of syncytia when tested against hMPV (Deffrasnes et al., 2008). The structure of this peptide suggests the interruption of fusion mechanism. More specifically, HRA and HRB rearrange the stable beam of six helices, a phenomenon that leads to the fusion of the membranes through the placement of the N-terminal of the fusion peptide next to the transmembrane C-terminal domain of the protein (Yin et al., 2005; Melero and Mas, 2015). Previous research suggests that the mechanism of action of the HRA2pl peptide is through its interaction with HRB when the F protein is refolded at the post-fusion state, which can result in a deficient fusion process. The HRB sequence is FNVALDQVFESIENSQALVDQSNRILSSAE.

In this study, we tested the antiviral activity of HRA2 expressed in *Nicotiana benthamiana* plants by a transient expression system (Márquez-Escobar et al., 2015). The effect of the fusion peptide was evaluated with an entry assay, while the viral titer and the size of the syncytium were determined with or without the peptide treatment. According to our data we could suggest that the HRA2pl peptide diminish the process of fusion for the entry of the virus and a 4-log decrease in the viral titer of the hRSV, hMPV, and hPIV-2 strains. These findings led us to study the effectiveness of HRA2pl on viral isolates from clinical samples of respiratory infection, where a 50% reduction in the size of the syncytium was found.

## 2 Materials and methods

### 2.1 Cells and viruses

Human laryngeal epithelial type 2 cells (HEp-2; ATCC CCL23, USA, which reported a contamination with HeLa cells) (Galvan et al, 2014) and Vero cells (normal adult African green monkey kidney cells; ATCC CCL81) were used to multiply the viral stocks for the viral titer test. They were propagated as described in Payment and Trudel (1993). The viruses hRSV Long [subgroup A (hRSV-A; ATCC^®^ VR-26™)], hRSV 18537 [subgroup B (hRSV-B; ATCC^®^ VR-1400™)], hPIV-2 (ATCC^®^ VR-92™), and hMPV were isolated from clinical samples in our laboratory (Cerezo et al., 2016) The procedures for propagating the viruses and assessing the viral infectivity are described in Payment and Trudel (1993).

### 2.2 Clinical samples

The samples were retrieved from a bank of pharyngeal and nasopharyngeal specimens that were obtained from pediatric patients (0–14 years) with an acute respiratory infection, between August 30, 2004, and February 13, 2014. The viral isolates obtained from the clinical samples were tested by endpoint RT-PCR as described in Perezbusta-Lara et al., 2020b. All subjects provided informed consent, and the study was reviewed and approved by the Research and Ethics Committee of the Faculty of Medicine, National Autonomous University of Mexico (UNAM; 089/2014; registry code: FMED/CI/SPLR/134/2014).

### 2.3 Fusion recombinant peptide HRA2pl

The sequence was based on Deffrasnes et al. (2008). The construction of the HRA2pl recombinant peptide (viral vector pICH11599 with the HRA2pl peptide coding sequence: HHHHHHSSGLVPRGSMKETAAAKFERQ HMDSPDLGTDDDDKAMADI GSEFENLYQGAKTIRLESEVTAIKNALKKTNEAVSTLGNGVRVLATAVRELKDFVSKN) and other two vectors required for the transient transformation (viral vector pICH4851 with the RNA-dependent polymerase (RdRp) coding sequence and the viral vector pICH10881 with the integrase coding sequence). were made by Veronica Marquez Escobar (Ph.D.). Six-week-old *N. benthamiana* plants were transiently transformed to express HRA2pl, which was subsequently purified by affinity chromatography with imidazole gradient through a zinc column. Two samples, B1 (172.31 ng/µL, imidazole 500 mM) and B2 (182.9 ng/µL, imidazole 250 mM), were provided by the Alpuche Solís Laboratory (Instituto Potosino de Investigación Científica y Tecnológica) for the fusion assays.

### 2.4 Cytotoxicity assay

The cytotoxic effect of the fusion peptide HRA2pl on HEp-2 and Vero cells was determined using AlamarBlue protocol as described elsewhere (O’Brien et al., 2000). Briefly, confluent monolayers of HEp-2 cells (1 × 10^5^ cells per well) or Vero cells (1 × 10^5^ cells per well) were seeded into 96-well culture plates and were incubated overnight at 37°C under 5% CO_2_.) and were cultured overnight with DMEM containing 5% of fetal bovine serum (FBS) at 37 and °C under 5% CO_2_. For the kinetics of cell viability (0 h, 24 h and 48 h) the medium with 5% FBS was removed, and HRA2pl (B1: 172.31 ng/μL; B2: 182.9 ng/μL), were applied in the presence of fresh DMEM without serum. Simultaneously, AlamarBlue reagent was added. The kinetics of cell viability was evaluated by spectrophotometry at 0 h, 24 h, and 48 h. The control of cell viability assay were done in parallel for both cell lines (Hep-2 cells or Vero cells), that were cultured with DMEM without serum and without peptide. The assays were conducted in triplicate (i.e., three experiments per assay). The kinetics of cell viability was evaluated by spectrophotometry at 0 h, 24 h, and 48 h. The absorbance was read at 570 nm. The viability was calculated as follows:


$$Cell\;viability=\frac{absorbance\;with\;treatment}{absorbance\;without\;treatment}\;\times 100$$

### 2.5 Determination of the optimal concentration of HRA2pl

The optimal concentration of the fusion peptides HRA2pl B1 and B2 was determined by measuring the reduction of the viral titer expressed as TCID_50_/ml (Payment and Trudel, 1993). Confluent HEp-2 and Vero cell monolayers were treated with different concentrations of HRA2pl B1 or B2 (3.2 μg/ml, 2.1 μg/ml, and 1.8 μg/ml) against hRSV (1.9 × 10^4^ TCID_50_/ml), hMPV (clinically isolated), or hPIV-2 (1 × 10^5^ TCID_50_/ml). The treated cell monolayers were incubated with the mixtures previously described for 4 h at 4°C to avoid virus internalization. Afterwards, the 96-well culture plates were incubated for 2 h at 37°C under 5% CO_2_ to allow the internalization of viral particles. Subsequently, the treated cultures were incubated for 72 h at 37°C (Lupini et al., 2009; Chang et al., 2013; Visintini Jaime et al., 2013; Lelešius et al., 2019) and the effect of HRA2pl on the viral titer was determined by the TCID50 assay (Payment and Trudel, 1993). The outcome of the HRA2pl treatment against hMPV was studied by measuring the size and number of the distinctive syncytia, corresponding to the cytopathic effect (CPE). To visualize CPE, the monolayers were fixed with methanol for 5 min. To stain the cells, the solution of crystal violet was added for 4 min at room temperature with constant stirring. The stain solution was removed, and the plate was rinsed with running water. The size of the syncytium was measured with the software ImageJ 1.5b. The assays were conducted in triplicate (i.e., three experiments per assay).

### 2.6 Entry assay

To test the virucidal activity of HRA2pl, we evaluated its effect on viral entry with the TCID50 assay for the hRSV and hPIV-2 strains or the reduction of CPE for the hMPV (Lupini et al., 2009; Chang et al., 2013; Visintini Jaime et al., 2013; Lelešius et al., 2019). Briefly, HEp-2 or Vero cells were grown in 96-well culture plates and incubated for 24 h under 5% CO_2_. The confluent monolayers were treated with a mixture of hRSV (1.9 × 10^4^ TCID_50_/ml), hMPV (virus isolated from clinical samples), or hPIV-2 (1 × 10^5^ TCID_50_/ml) and 3.2 μg/ml of HRA2pl. Then, the treated cell monolayers were incubated with the mixtures previously described for 4 h at 4°C to avoid virus internalization. Subsequently, the plates were incubated with the mixture described above for 2 h at 37°C under 5% CO_2_ to allow the internalization of viral particles. Next, the inoculum was removed, and 150 μl of fresh DMEM (without serum) per well were added for 48 h at 37°C under 5% CO_2_. Subsequently, the viral titer was determined by the TCID50 assay as described elsewhere (Payment and Trudel, 1993) For hMPV, the changes in CPE (size and number of the distinctive syncytia) were determined.

The virucidal activity of HRA2pl was tested against viral isolates from clinical samples of respiratory infections. Briefly, Hep-2 or Vero cells were grown in 96-well culture plates and incubated for 24 h under 5% CO_2_. The 96 plates were divided into two sections: one was infected with the viral isolates without treatment with the peptide, and the other one was treated with both the viral isolates and 3.2 μg/ml of HRA2pl. The plates were then incubated at 4°C for 4 h. The assay followed the steps that are previously described in the above section. The effect of HRA2pl on the clinical samples was calculated as the change in the size and/or the number of distinctive syncytia in 10 microscopic fields per clinical sample. The controls of this assay were a mixture of DMEM and HRA2pl, DMEM and clinical viral isolates, and DMEM and cells. These controls were used for the statical analysis described in section 2.7.

### 2.7 Statistical analysis

The data were analyzed with one-way analyses of variance (ANOVA) and Student’s t-test. The differences among the mean values were tested for significance using the Tukey test. All results were considered significant when *p* ≤ 0.05. The data were analyzed and visualized using the statistical software GraphPad Prism 5.0.

## 3 Results

### 3.1 HRA2pl peptides did not have a cytotoxic effect on HEp-2 and Vero cells

**Figure 1:**
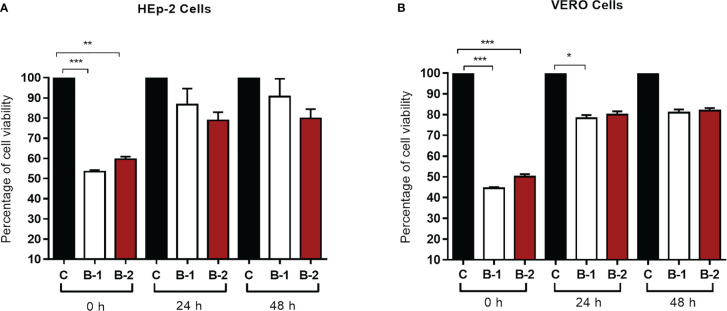
The HRA2pl peptide does not affect cell viability of **(A)** HEp-2 and **(B)** Vero cultures. For the assay, a confluent monolayer of HEp-2 and Vero cells (95%–100%) was treated with 90 μL of HRA2pl purified in DMEM (B1: 172.31 ng/μL; B2: 182.9 ng/μL) and 10 μL of AlamarBlue to measure the absorbance at 0 h, 24 h, and 48 h. *NS*: Nonsignificant; *p< 0.05;**p< 0.01; ***p< 0.001.

Before evaluating B1 and B2 HRA2pl’s antiviral activity, the peptide’s innocuity on the HEp-2 and Vero cells was examined with a 48-h kinetics of cytotoxicity assay. B1 and B2 were not cytotoxic according to the measurement of cell viability, independently of the peptide’s concentration (**Figure 1**). About our data, particularly the time 0 h of the kinetic cell viability assay, showed a decrease in the cell viability, result that took our attention. So, we tried to explain this result considering that the assay was done in deprivation of serum and this factor could affect the cell proliferation, considering that, the previous 24 h, we cultured both cell lines with DMEM containing 5% of FBS. The peptide did not affect the cell viability because the cell lines recover their proliferation for the following points of the kinetics. It is important to consider that besides the deprivation of serum (claimed before) the cell monolayers probably lose some cells during the development of the assay because the monolayer was washed before we began the peptide treatment.

### 3.2 HRA2pl reduced the viral titer of hRSV and hPIV-2

**Figure 2:**
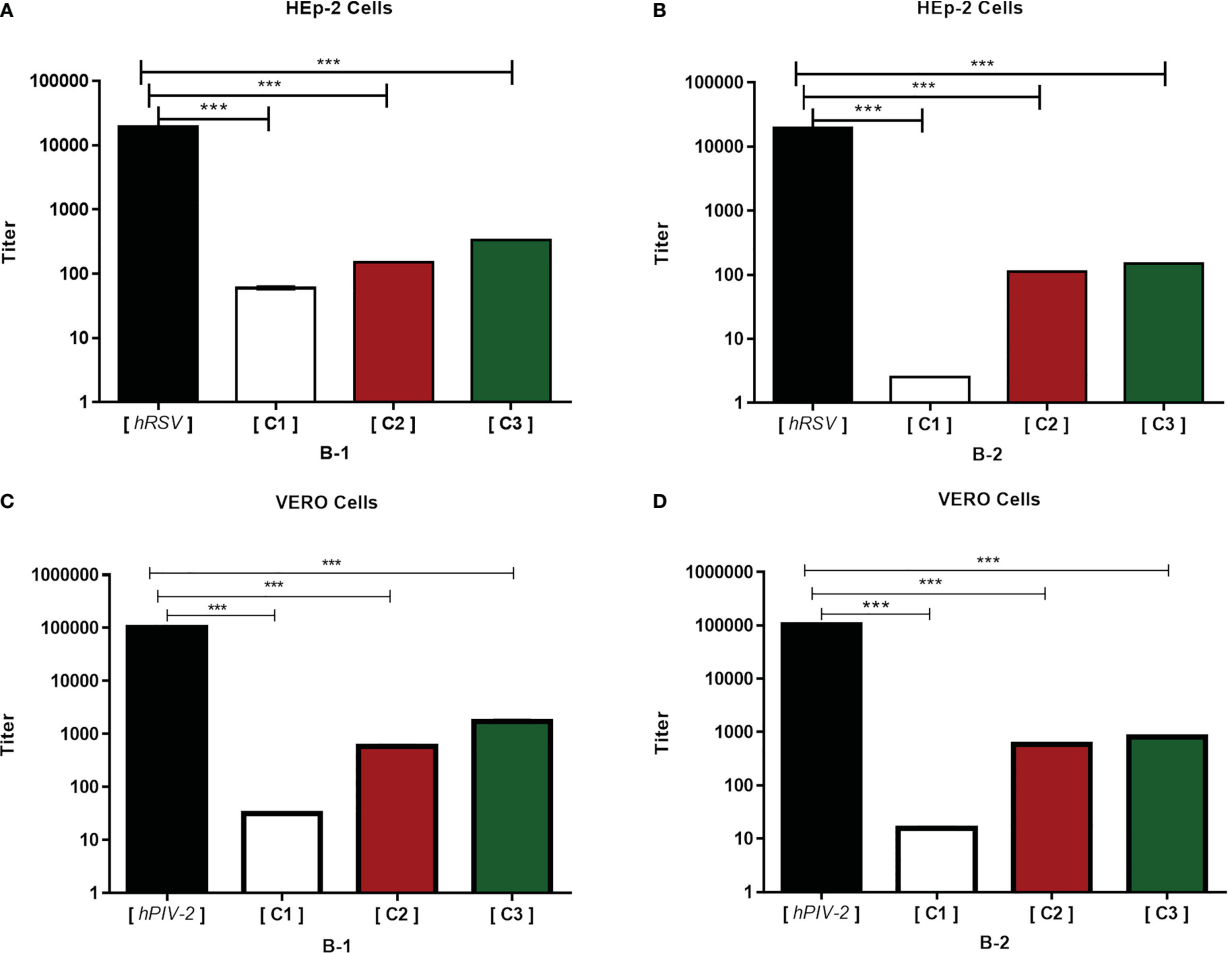
HRA2pl reduces the viral titer. For the assay, a confluent monolayer of HEp-2 **(A, B)** and Vero **(C, D)** cells (95%–100%) was treated with HRA2pl purified in DMEM at three different concentrations (C1: 3.2 µg/mL; C2: 2.1 µg/mL; C3: 1.8 µg/mL). The monolayers were treated with a mixture of C1, C2, or C3 and hRSV (1.9 × 10^4^ TCID_50_/ml) and hPIV-2 (1 × 10^5^ TCID_50_/ml). The reduction in the viral titer was established by TCID_50_/ml.***p< 0.001.

The antiviral effect of HRA2pl against hRSV and hPIV-2 was measured as the reduction of the viral titer. Three different concentrations of purified B1 or B2 (3.2 μg/ml, 2.1 μg/ml, 1.8 μg/ml) were tested. The viral titer of hRSV and hPIV-2 decreased at all tested concentrations. However, the most prominent reduction in viral production was observed at the concentration of 3.2 μg/ml of either B1 or B2. In the case of hRSV, the viral titer reduced from 1.9 × 10^4^ to 5.9 × 10^1^ TCID_50_/ml (**Figure 2A**) when applying B1. As for B2, the viral production reduced from 1.9 × 10^4^ to 2.5 TCID_50_/ml (**Figure 2B**). Similarly, in the case of hPIV-2, the B1 peptide reduced the viral titer from 1 × 10^5^ to 3.1 × 10^1^ TCID_50_/ml (**Figure 2C**), and the B2 peptide reduced it from 1 × 10^5^ to 1.5 × 10^1^ TCID_50_/ml (**Figure 2D**). Thus, the HRA2pl B2 peptide had the best antiviral activity against the production of viral particles of hRSV and hPIV-2.

### 3.3 HRA2pl treatment reduced the syncytium size of clinical isolates

**Figure 3:**
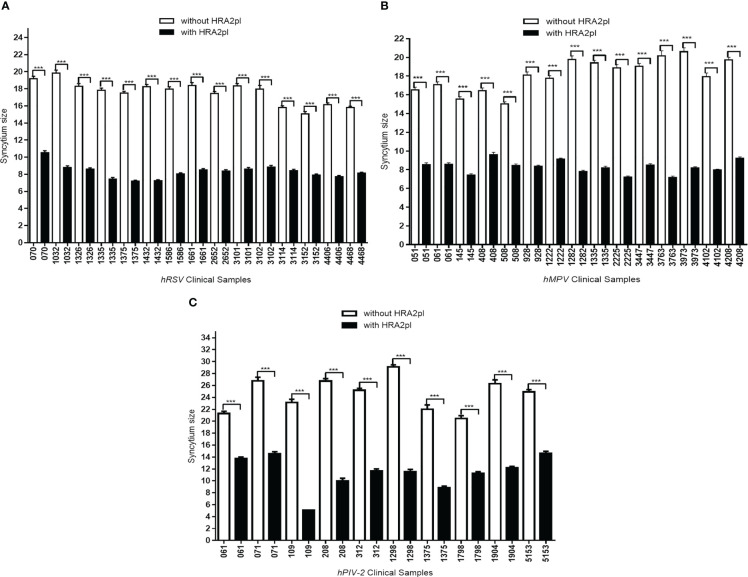
The syncytium size was reduced after treatment with HRA2pl B2. In HEp-2 cultures infected with **(A)** hRSV or **(B)** hMPV viral isolates, and **(C)** in Vero cultures infected with hPIV-2 viral isolates, treatment with HRA2pl significantly reduced the syncytium size. ***p< 0.001.

To test the peptide’s effect on pediatric patients’ samples, 40 clinical viral isolates, a representative sample of our viral bank, were studied. HRA2pl B2 treatment induced the reduction of the size of the syncytium. After treatment with the peptide, the hRSV-positive samples’ syncytium size reduced to 52.87% of the control syncytium size (**Figure 3A**). The size of the syncytium without treatment ranged from 15.05 mm to 19.83 mm (average: 17.59 mm). Meanwhile, treatment with the peptide produced a reduction in the size of the syncytium, reaching a size that ranged from 7.20 mm to 10.52 mm (average: 8.29 mm). A similar result was observed on the clinical samples typified as hMPV-positive. They showed a reduction of 54.33% compared to the samples without treatment (**Figure 3B**). The average syncytium size without treatment was 18.13 mm, while after treatment, it reached 8.28 mm. Finally, the effect of the peptide on the hPIV-2 clinical samples showed a reduction of the syncytium (53.88% of the control samples) (**Figure 3C**). The average size without treatment was 24.59 mm, which reached 11.34 mm after treatment with the peptide. Therefore, the HRA2pl peptide acts as an inhibitor of viral fusion by competition against the respiratory viruses hRSV, hMPV, and hPIV-2.

### 3.4 HRA2pl treatment modified the number of syncytia

**Figure 4:**
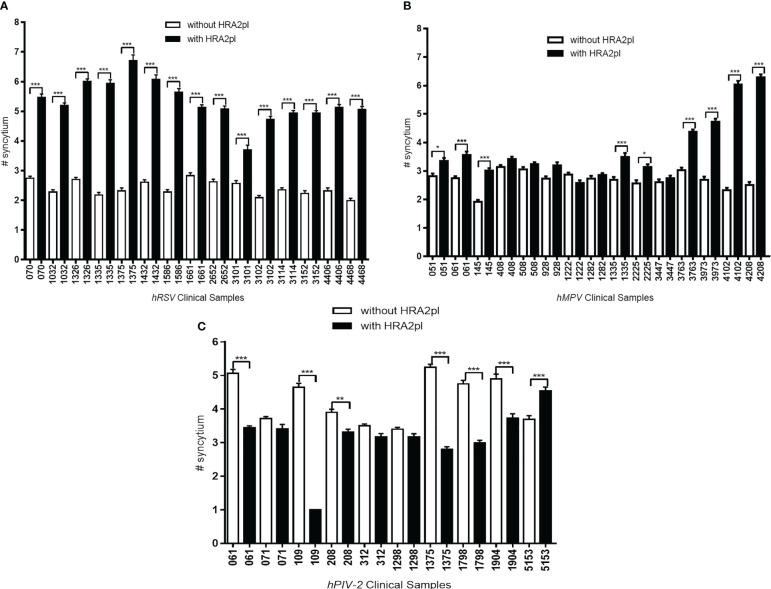
The changes in the number of syncytia after HRA2pl B2 treatment varied according to the viral infection. HRA2pl treatment increased the number of syncytia in **(A)** HEp-2 cells infected with hRSV viral isolates. **(B)** Some, but not all, HEp-2 cell cultures infected with hMPV viral isolates showed an increased number of syncytia after treatment with HRA2pl. **(C)** Treatment with the HRA2pl peptide decreased the number of syncytia in Vero cells infected with hPIV-2 viral isolates. NS, Nonsignificant; *p< 0.05; **p< 0.01; ***p< 0.001.

Even though treatment with HRA2pl caused a significant reduction in the size of the syncytium, an increase in the number of syncytia was observed. This was probably associated with deficient fusion resulting from the competition between the viral fusion protein of the viral isolates versus the HRA2pl fusion peptide. In the case of the hRSV-positive samples, an increase from 2.41 to 5.31 syncytia per field was found after treatment with the peptide (**Figure 4A**). The hMPV samples presented an increase from 2.69 to 3.73 syncytia per field after treatment (**Figure 4B**). Nonetheless, a reduction in the number of syncytia per field was recorded in the case of hPIV-2 clinical samples (without treatment: 4.28 syncytia per field; with treatment: 3.16 syncytia per field; **Figure 4C**).

## 4 Discussion

In this study, we demonstrated that the HRA2pl peptide efficiently blocks the viral fusion of hRSV, hMPV, and hPIV-2 in an *in vitro* model, resulting in the reduction of the syncytium size. Moreover, we proved that the HRA2pl peptide reduced the number of syncytia in the hPIV2-positive clinical samples, contrary to the hRSV- or hMPV-positive samples. As for the latter, although the number of syncytia was not reduced, the reduction in their size is a noteworthy biological event that indicates the antiviral potential of HRA2pl in clinical samples.

The global presence of the hRSV, hMPV, and hPIV-2 viruses that are responsible for respiratory diseases, impacts the health of thousands of children and adults. Despite the relevance of viral ARTIs as a global persistent public health problem (Lu et al., 2013b) and the subsequent high risk of morbidity and mortality (Taylor, 2017), no prophylactic vaccines or effective antiviral treatments against them are currently available, with those applied providing only limited protection (Takeuchi and Akira, 2009). The production of a safe, efficient, and low-cost treatment against these viruses is a challenge to be faced.

The HRA2pl peptide inhibits the *in vitro* entry of hMPV at the fusion stage (Márquez-Escobar et al., 2015). In this context, we aimed to study whether HRA2pl can inhibit the entrance of not only hMPV but also other human respiratory viruses, like hRSV or hPIV-2, at the fusion stage. Our results demonstrate that the HRA2pl peptide produced a reduction in the size of the syncytium in all clinical samples independently of the type of respiratory viruses. This is in line with the fact that F proteins are phylogenetically close to each other (Russell et al., 2003) and are conserved across the families of *Pneumoviridae* and *Paramyxoviridae* (Aggarwal and Plemper, 2020).

F proteins are synthesized as an F0 precursor without fusion activity; their transition from F0 to F1–F2 is produced by the host cell’s furin-type proteases residing in the Golgi apparatus (Aggarwal and Plemper, 2020). The F1 subunit contains the fusion peptide, a domain of hydrophobic amino acids, and the highly conserved amphipathic α-helical regions with 3–4 HR patterns adjacent to the fusion peptide, called the HRA and HRB domains (Aggarwal et al., 2020). Previous research showed that the recombinant HRA peptides were more efficient than HRB in inhibiting the fusion of hMPV and the virus’ entry to the host cell, even at low concentrations (Deffrasnes et al., 2008). Here, we conclude that the HRA2pl decreased the size of the syncytium of hRSV, hMPV, and hPIV-2 viral isolates by up to 50% in HEp-2 and Vero cells.

Our findings contribute to the literature concerning the therapeutic effects of interference with viral fusion proteins, paving the way toward clinical trials.

## Data availability statement

The raw data supporting the conclusions of this article will be made available by the authors, without undue reservation.

## Ethics statement

The study was conducted in accordance with the bi-osafety regulations of the World Medical Association’s Declaration of Helsinki regarding the ethical conduct of research involving humans and was approved by the Research Ethics Board of Facultad de Medicina, UNAM (project number 101-2012). The informed consent letters were approved by the Committee of Ethics and Research of the División de Investigación de la Facultad de Medicina de la UNAM (FMED/CI/SPLR/004/2016).

## Author contributions

Conceptualization, RT, UC and MS. Methodology, UC, NP, LC and MS. Validation UC, NP, LC and MS. Formal analysis, RT, UC and MS. Investigation, UC and RT. Resources, JH. Data curation, RT, UC and MS. Statistical analysis, UC, and LC. Writing, MS and RT. Supervision, RT and JH Project administration, JH, and RT. Funding acquisition, JH. All authors contributed to the article and approved the submitted version. JH passed away by COVID a year ago.

## References

[B1] Aggarwal M. Plemper R. K. (2020). Structural insight into paramyxovirus and pneumovirus entry inhibition. Viruses. 12 (3), 342. doi: 10.3390/v12030342 32245118 PMC7150754

[B2] Allander T. Tammi M. T. Eriksson M. Bjerkner A. Tiveljung-Lindell A. Andersson B. (2005). Cloning of a human parvovirus by molecular screening of respiratory tract samples. PNAS. 102 (36), 12891–12896. doi: 10.1073/pnas.0504666102 16118271 PMC1200281

[B3] Berry M. Gamieldien J. Fielding B. (2015). Identification of new respiratory viruses in the new millennium. Viruses. 7 (3), 996–1019. doi: 10.3390/v7030996 25757061 PMC4379558

[B4] Bicer S. Giray T. Çöl D. Erdağ G. I. Vitrinel A. Gürol Y.. (2013). Virological and clinical characterizations of respiratory infections in hospitalized children. Ital. J. Pediatr. 39 (1), 22. doi: 10.1186/1824-7288-39-22 23536956 PMC3621398

[B5] Cerezo L. G. Zárate C. K. Alpuche-Lazcano S.. (2016). Diagnóstico molecular para la detección de metapneumovirus humano a partir de aislados virales en pacientes pediátricos con infección respiratoria aguda. Investigación en Discapacidad 5 (2), 88–95.

[B6] Chang J. S. Wang K. C. Yeh C. F. Shieh D. E. Chiang L. C. (2013). Fresh ginger (Zingiber officinale) has anti-viral activity against human respiratory syncytial virus in human respiratory tract cell lines. J. Ethnopharmacol. 145 (1), 146–151. doi: 10.1016/j.jep.2012.10.043 23123794

[B7] Deffrasnes C. Hamelin M. E. Prince G. A. Boivin G. (2008). Identification and evaluation of a highly effective fusion inhibitor for human metapneumovirus. Antimicrob. Agents Chemother. 52 (1), 279–287. doi: 10.1128/aac.00793-07 17967906 PMC2223880

[B8] Galvan M. A. Cabello C. Mejia F. Valle L. Valencia E. Manjarrez M. E. (2014). Parainfluenza virus type 1 induces epithelial IL-8 production *via* p38-MAPK signalling. J. Immunol. Res. 2014, 1–12. doi: 10.1155/2014/515984 PMC4072021 25013817

[B9] García-García M. L. Calvo C. Rey C. Díaz B. Molinero M. D. M. Pozo F.. (2017). Human metapnuemovirus infections in hospitalized children and comparison with other respiratory viruses. 2005-2014 prospective study. PloS One 12 (3), e0173504. doi: 10.1371/journal.pone.0173504 28301570 PMC5354294

[B10] Lelešius R. Karpovaitė A. Mickienė R. Drevinskas T. Tiso N. Ragažinskienė O.. (2019). *In vitro* antiviral activity of fifteen plant extracts against avian infectious bronchitis virus. BMC Vet. Res. 15 (1), 178. doi: 10.1186/s12917-019-1925-6 31142304 PMC6540435

[B11] Lu Y. Wang S. Zhang L. Xu C. Bian C. Wang Z.. (2013b). Epidemiology of human respiratory viruses in children with acute respiratory tract infections in jinan, China. Clin. Dev. Immunol. 2023, 1–8. doi: 10.1155/2013/210490 PMC3865640 24363757

[B12] Lupini C. Cecchinato M. Scagliarini A. Graziani R. Catelli E. (2009). *In vitro* antiviral activity of chestnut and quebracho woods extracts against avian reovirus and metapneumovirus. Res. Vet. Sci. 87 (3), 482–487. doi: 10.1016/j.rvsc.2009.04.007 19435637

[B13] Madhi S. A. Polack F. P. Piedra P. A. Munoz F. M. Trenholme A. A. Simões E. A.. (2020). Respiratory syncytial virus vaccination during pregnancy and effects in infants. NEJM. 383 (5), 426–439. doi: 10.1056/nejmoa1908380 32726529 PMC7299433

[B14] Manzoni P. Figueras-Aloy J. Simões E. A. F. Checchia P. A. Fauroux B. Bont L.. (2017). Defining the incidence and associated morbidity and mortality of severe respiratory syncytial virus infection among children with chronic diseases. Infect. Dis. Ther. 6 (3), 383–411. doi: 10.1007/s40121-017-0160-3 28653300 PMC5595774

[B15] Márquez-Escobar V. A. Tirado-Mendoza R. Noyola D. E. Gutiérrez-Ortega A. Alpuche-Solís N. G. (2015). HRA2pl peptide: a fusion inhibitor for human metapneumovirus produced in tobacco plants by transient transformation. Planta. 242 (1), 69–76. doi: 10.1007/s00425-015-2277-5 25828350

[B16] Melero J. A. Mas V. (2015). The pneumovirinae fusion (F) protein: A common target for vaccines and antivirals. Virus Res. 209, 128–135. doi: 10.1016/j.virusres.2015.02.024 25738581

[B17] Morris S. K. Dzolganovski B. Beyene J. Sung L. (2009). A meta-analysis of the effect of antibody therapy for the prevention of severe respiratory syncytial virus infection. BMC Infect. Dis. 9 (1), 106. doi: 10.1186/1471-2334-9-106 19575815 PMC2720977

[B18] Muňoz F. M. Swamy G. K. Hickman S. P. Agrawal S. Piedra P. A. Glenn G. M.. (2019). Safety and immunogenicity of a respiratory syncytial virus fusion (F) protein nanoparticle vaccine in healthy third-trimester pregnant women and their infants. J. Infect. Dis. 220 (11), 1802–1815. doi: 10.1093/infdis/jiz390 31402384

[B19] O’Brien K. L. Chandran A. Weatherholtz R. Jafri H. S. Griffin M. P. Bellamy T.. (2015). Efficacy of motavizumab for the prevention of respiratory syncytial virus disease in healthy native American infants: a phase 3 randomised double-blind placebo-controlled trial. Lancet Infect. Dis. 15 (12), 1398–1408. doi: 10.1016/s1473-3099(15)00247-9 26511956

[B20] O’Brien J. Wilson I. Orton T. Pognan F. (2000). Investigation of the alamar blue (resazurin) fluorescent dye for the assessment of mammalian cell cytotoxicity. Eur. J. Biochem. 267 (17), 5421–5426. doi: 10.1046/j.1432-1327.2000.01606.x 10951200

[B21] Özgür Ç. Demet Ç. (2021). Viral respiratory tract pathogens during the COVID-19 pandemic. Eurasian J. Med. 53 (2), 123–126. doi: 10.5152/eurasianjmed.2021.20459 34177295 PMC8184047

[B22] Payment P. Trudel M. (1993). ‘Isolation and identification of viruses’ in *Methods and techniques in virology.* N.Y. USA: Marcel Dekker Inc pp, 309–310. doi: 10.1002/rmv.1980040308

[B23] Perezbusta-Lara N. Tirado-Mendoza R. Ambrosio-Hernández J. R. (2020b). Respiratory infections and coinfections: geographical and population patterns. Gaceta México. 156 (4), 263–269. doi: 10.24875/gmm.m20000396 32831337

[B24] Pierangeli A. Scagnolari C. Antonelli G. (2018). Respiratory syncytial virus in infants. Minerva Pediatr. 70 (6), 553–565. doi: 10.23736/S0026-4946.18.05312-4 30334622

[B25] Price R. H. M. Graham C. Ramalingam S. (2019). Association between viral seasonality and meteorological factors. Sci. Rep. 9 (1), 8–11. doi: 10.1038/s41598-018-37481-y 30700747 PMC6353886

[B26] Rodríguez P. E. Frutos M. C. Adamo M. P. Cuffini C. Cámara J. A. Paglini M. G.. (2020). Human metapneumovirus: Epidemiology and genotype diversity in children and adult patients with respiratory infection in córdoba, Argentina. PloS One 15 (12), e0244093. doi: 10.1371/journal.pone.0244093 33370354 PMC7769284

[B27] Russell C. J. Kantor K. L. Jardetzky T. S. Lamb R. A. (2003). A dual-functional paramyxovirus f protein regulatory switch segment. JCB. 163 (2), 363–374. doi: 10.1083/jcb.200305130 14581458 PMC2173521

[B28] Schwarze J. (2010). Respiratory viral infections in infants: Causes, clinical symptoms, virology, and immunology. CMR. 23 (1), 47–98. doi: 10.1128/cmr.00032-09 PMC2806659 20065326

[B29] Shachor-Meyouhas Y. Ben-Barak A. Kassis I. (2011). Treatment with oral ribavirin and IVIG of severe human metapneumovirus pneumonia (HMPV) in immune compromised child. Pediatr. Blood Cancer 57 (2), 350–351. doi: 10.1002/pbc.23019 21671370

[B30] Shang Z. Tan S. Ma D. (2021). Respiratory syncytial virus: from pathogenesis to potential therapeutic strategies. Int. J. Biol. Sci. 17 (14), 4073–4091. doi: 10.7150/ijbs.64762 34671221 PMC8495404

[B31] Takeuchi O. Akira S. (2009). Innate immunity to virus infection. Immunol. Rev. 227 (1), 75–86. doi: 10.1111/j.1600-065x.2008.00737.x 19120477 PMC5489343

[B32] Taylor G. (2017). Animal models of respiratory syncytial virus infection. Vaccine. 35 (3), 469–480. doi: 10.1016/j.vaccine.2016.11.054 27908639 PMC5244256

[B33] Visintini Jaime M. F. Redko F. Muschietti L. V. Campos R. H. Martino V. S. Cavallaro L. V. (2013). *In vitro* antiviral activity of plant extracts from asteraceae medicinal plants. Virol. J. 10 (1), 245. doi: 10.1186/1743-422x-10-245 23890410 PMC3733733

[B34] Yang J. Hillson E. Mauskopf J. Copley-Merriman C. Shinde V. Stoddard J. (2017). The epidemiology of medically attended respiratory syncytial virus in older adults in the united states: A systematic review. PloS One 12 (8), e0182321. doi: 10.1371/journal.pone.0182321 28797053 PMC5552193

[B35] Yin H. S. Paterson R. G. Wen X. Lamb R. A. Jardetzky T. S. (2005). Structure of the uncleaved ectodomain of the paramyxovirus (hPIV3) fusion protein. Proc. Natl. Acad. Sci. U. S. A. 102 (26), 9288–9293. doi: 10.1073/pnas.0503989102 15964978 PMC1151655

[B36] Zappa A. Perin S. Amendola A. Bianchi S. Pariani E. Ruzza M. L.. (2008). Epidemiological and molecular surveillance of influenza and respiratory syncytial viruses in children with acute respiratory infections, (2004/2005 season). Microbiologia Medica. 23 (1), 7–21. doi: 10.4081/mm.2008.2592

